# Comparison of functional performance outcomes between oral patented crystalline glucosamine sulfate and platelet-rich plasma among knee osteoarthritis patients: a propensity score matching analysis

**DOI:** 10.1007/s40520-024-02814-7

**Published:** 2024-08-10

**Authors:** Chavarin Amarase, Aree Tanavalee, Srihatach Ngarmukos, Chotetawan Tanavalee, Nonn Jaruthien, Pakpoom Somrak, Saran Tantavisut

**Affiliations:** 1 Biologics for Knee Osteoarthritis Research Unit, Faculty of Medicine, Chulalongkorn University, Bangkok, Thailand; 2 Department of Orthopaedics, Faculty of Medicine, Chulalongkorn University and King Chulalongkorn Memorial Hospital, Thai Red Cross Society, 1873 Rama IV Road, Bangkok, 10330 Thailand

**Keywords:** Functional performance, Platelet-rich plasma, PRP, Glucosamine, Knee, Osteoarthritis, OA

## Abstract

**Background:**

Among the medications used to treat knee osteoarthritis (OA), oral patented crystalline glucosamine sulfate (pCGS) and platelet-rich plasma (PRP) have become popular alternatives to painkillers or nonsteroidal anti-inflammatory drugs (NSAIDs). Although studies have shown that pCGS and PRP improve clinical outcomes, no study has compared outcomes between these optional treatments. We compared functional performance outcomes from baseline to the 1-year follow-up (FU) between oral pCGS and PRP in patients with knee OA.

**Materials and methods:**

Three hundred eighty-two patients receiving oral pCGS and 122 patients receiving PRP injections were enrolled for a review of functional performance outcomes, including a five-time sit-to-stand test (5xSST), time up-and-go test (TUGT), and 3-minute walk distance test (3MWDT). The patients were followed up for one year. The pCGS group received 1500 mg daily, whereas the PRP group received 2 cycles of intra-articular injections at week 0 and week 6. Using propensity score matching based on age, sex, height, weight, BMI, and Kellgren and Lawrence (KL) classification, all three functional performance outcomes were compared between the baseline (pretreatment), 6-week, 12-week, 24-week, and 1-year FUs.

**Results:**

With a ratio of 2:1 (pCGS: PRP), 204 patients in the pCGS group were matched with 102 patients in the PRP group. Compared with the baseline levels, the PRP group showed significant improvements in 5xSST and TUGT outcomes from 6 weeks and significant improvements in 3MWDT outcomes from 12 weeks, whereas the pCGS group showed significant improvements in TUGT outcomes from 6 weeks and significant improvements in 5xSST and 3MWDT outcomes from 12 weeks. At the 24-week and 1-year FU, both groups showed significant improvements in all three functional performance tests without adverse events.

**Conclusions:**

Although the PRP group showed faster improvements in 5xSST outcomes at six weeks, from the 12-week to 1-year FU, both the pCGS and PRP groups showed significant improvements in 5xSST, TUGT, and 3MWDT outcomes. As the use of PRP is more complicated and invasive than the use of oral pCGS, the benefits and drawbacks of selecting PRP over pCGS in knee OA treatment should be examined.

## Introduction

Knee osteoarthritis (OA) is a degenerative disease that affects the cartilage around the knee. The symptoms of knee OA include pain, limited joint motion, difficulty walking, and decreased daily activities that affect quality of life [1, 2]. According to data from the Medical Expenditure Panel Survey (MEPS) from 2008 to 2010, an estimated 14.4 to 51.4 million osteoarthritis (OA) cases occur annually, with prevalence rates ranging from 6.3 to 22.4% each year [3]. There are several methods for treating knee OA, including controlling body weight, patient education, quadriceps strengthening exercises, lifestyle modification, rehabilitation, knee support, oral analgesic drugs, topical analgesic drugs, oral nonsteroidal anti-inflammatory drugs (NSAIDs), topical NSAIDs, symptomatic slow-acting drugs for OA (SYSADOA), intra-articular injection of steroids, hyaluronic acid, platelet-rich plasma (PRP) and surgical intervention [3–5]. The treatment choice depends on several factors, including symptoms related to the severity of knee OA (as determined by the Kellgren and Lawrence (KL) classification [6]), comorbidities, patient compliance, physician opinions, and patient perceptions related to drugs for treating knee OA.

Regarding medications for mild-to-moderate-several knee OA, patients who avoid the use of painkillers, anti-inflammatory drugs, and intra-articular medicines, which are considered more invasive treatments, may prefer to take joint supplements, such as glucosamine; in particular, the literature has shown that only patented crystalline glucosamine sulfate (pCGS) provides a positive benefit to patients [7–9]. On the other hand, patients avoiding any oral or intra-articular medications, as well as joint supplements, may prefer orthobiologic treatment, of which intra-articular platelet-rich plasma (PRP) injection is a common option that exhibits a biological mechanism to fight against the degenerative process of the joint [10].

Glucosamine, a water-soluble amino monosaccharide, is a complex glycosaminoglycan that is linked to aggrecans to form part of the cartilage matrix [11]. Since 2005, the Cochrane Review has demonstrated the effectiveness of glucosamine sulfate (GS) in reducing pain and improving function compared with a placebo in terms of mild anti-inflammatory properties [12]. A recent systematic review and meta-analysis revealed that GS effectively reduced pain and improved the Western Ontario and McMaster Universities Osteoarthritis Index (WOMAC) score in patients with knee OA [9]. Furthermore, several studies reported that compared with other forms of glucosamine, patented crystalline glucosamine sulfate (pCGS) was more effective at managing pain, slowing disease progression, and enhancing cost-effectiveness [13–15]. According to Kucharz EJ et al., the pCGS, compared with generic and over-the-counter formulations of GS, provides a highly bioavailable once daily dose (1500 mg) and consistently reaches the plasma levels of approximately ten µM required to inhibit interleukin-1-induced expression of genes involved in the pathophysiology of joint inflammation and tissue destruction [14]. In 2019, the European Society for Clinical and Economic Aspects of Osteoporosis, Osteoarthritis and Musculoskeletal Diseases (ESCEO) revised the algorithm recommendations for treating knee OA published in 2014, which strongly recommended using pCGS as part of a core set treatment comprising information access/education, weight loss, and an exercise program in step one for the treatment of knee OA [16].

PRP, a biological component derived from venous blood, has been an optional treatment for knee OA for more than ten years. Several studies have shown that intra-articular PRP injections improve clinical outcomes such as pain and WOMAC scores during short- and mid-term follow-up (FU) [17–20]. Despite positive outcomes, not all major guidelines for knee OA treatment have endorsed the use of PRP [21–23]. However, this optional orthobiologic treatment has spread worldwide over the past decade [24–26].

Both oral pCGS and intra-articular PRP injection have demonstrated benefits in treating knee OA, but a comparative study has not been performed. Therefore, the present study used propensity score matching to compare functional performance outcomes between oral pCGS and intra-articular PRP injection at the 1-year FU.

## Methods

This study was approved by our institutional review board (IRB) (COA No. 0894/2022 and IRB No. 0360/65). From January 2019 to January 2020, data from all outpatients diagnosed with knee OA were reviewed. The inclusion criteria were as follows: OA knee according to the American College of Rheumatology (ACR) [27], radiographic knee OA graded 1 to 4 on the basis of the Kellgren-Lawrence system [6], age between 45 and 85 years, body mass index (BMI) over 30 kg/m^2^, visual analogue scale (VAS) pain score greater than four during knee motion, arc of motion exceeding 90 degrees, coronal plane knee deformity of less than 10 degrees, and absence of skin lesions or signs of infection around the affected knee. The exclusion criteria were as follows: thrombocytopenia, inflammatory arthritis, prior knee surgery, and recent use of painkillers or nonsteroidal anti-inflammatory drugs (NSAIDs) within the past two weeks. Among the eligible patients, only those who received treatment with oral pCGS and intra-articular PRP injection were enrolled in the study.

### Treatment protocol

**Fig. 1:**
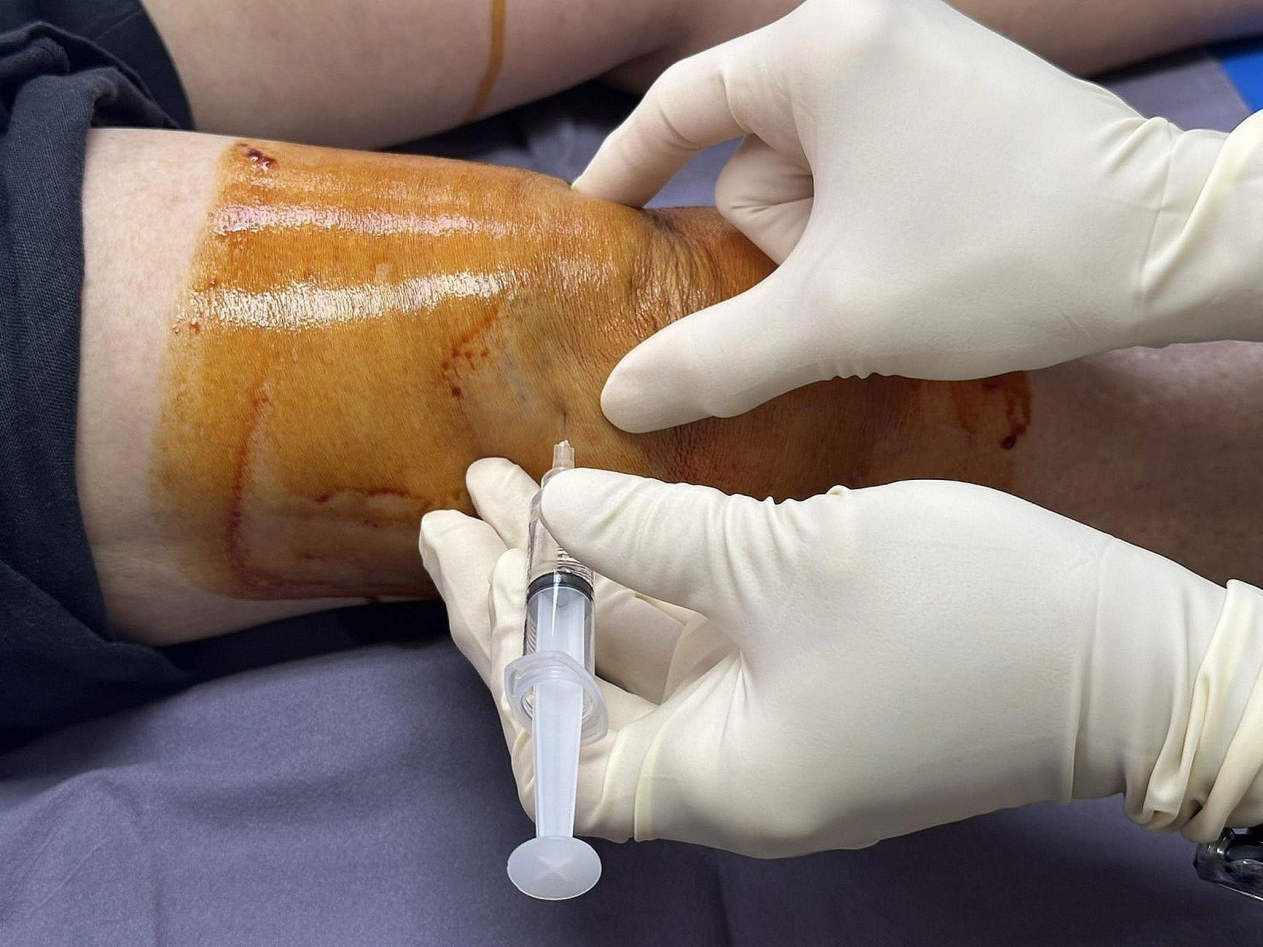
Intra-articular leukocyte-poor platelet-rich plasma (LP-PRP) injection via the superolateral direction of the knee under sterile conditions via a 24-gauge needle

For the Oral pCGS group, patients were prescribed Viartril 500 mg (Rottapharm Madaus, Confienza, Italy) and instructed to take three capsules once daily, totalling 1500 mg daily, before breakfast. For the PRP group, the preparation of leukocyte-poor PRP (LP-PRP) involved the collection of 30 mL of venous blood from the patient’s cubital vein, utilizing a blood collection tube containing anticoagulant citrate dextrose (ACD) solution (Vacuette, Greiner Bio-One, Austria). The collected blood was centrifuged twice, following the technique published by Perez et al. [28]. The initial centrifugation was conducted at 100 × g for 10 min to separate the contents of the tube into three distinct layers: the plasma, buffy coat, and erythrocyte layers. The plasma layer was subsequently carefully transferred to a new sterile tube, and a second centrifugation was performed at 400 × g for 8 min. Next, the upper two-thirds of the plasma content was removed, leaving the lower one-third of the plasma, which was then shaken to ensure the mixing of the platelet cells and plasma. This resulting mixture was designated LP-PRP. The remaining LP-PRP, approximately 5.5–7.0 mL, was supplemented with a 0.1% volume of CaCl_2_ to induce activation before injection. Under sterile conditions, the intra-articular LP-PRP injection was administered via the superolateral direction of the knee (Fig. 1). All knee OA patients in the PRP group were injected two times: at week 0 and at week 6.

### Functional performance tests

During the duration of knee OA treatment at the outpatient clinic, all patients included in the study were evaluated via three performance-based tests: the time up and go test (TUGT) [29], which measures the time it takes for patients to stand up from a chair, walk for 3 m with or without a walking aid, and then return to the initial seated position after completing a turn; the five-time sit-to-stand test (5xSST) [30], which measures the time is takes for patients to transition from a seated position in a chair to a fully upright standing position with their body erect and straight and then return to the seated position five times; and the three-minute walk distance test (3MWDT), which is a modified version of the 6-minute walk distance (6MWD) test [31] and measures the distance patients can walk within 3 min, with or without the use of a gait aid.

All knee OA patients in both groups underwent evaluation for functional performance outcomes, including the TUGT, 5xSST, and 3MWDT, at five timepoints: week 0, week 6, week 12, week 24, and 1 year. These evaluations were conducted by two investigators via a digital stopwatch. Baseline measurements for all functional performance outcomes were obtained at week 0. All functional performance outcomes were subsequently re-evaluated and compared to their respective baseline values. During the evaluation period, none of the patients received physical therapy, therapeutic exercise, or any other kind of treatment besides pCGS and PRP.

### Statistical analysis

All the data were analysed via IBM SPSS Statistics version 29 software. As the number of patients taking pCGS was greater than the number of patients taking PRP, the propensity score matching was performed using a ratio of 2:1 (pCGS: PRP). Matching was performed based on age, sex, height, weight, BMI, and KL classification. Qualitative data are presented as frequencies and percentages, whereas quantitative data are presented as the means ± standard deviations (SDs). The chi-square test was performed for categorical data. Repeated-measures ANOVA, followed by Tukey’s post hoc test, was conducted to evaluate parameters across the baseline and FU visits within each group. Independent t tests were used to assess differences between groups at each FU visit. A p value of < 0.05 was considered statistically significant.

## Results

**Fig. 2:**
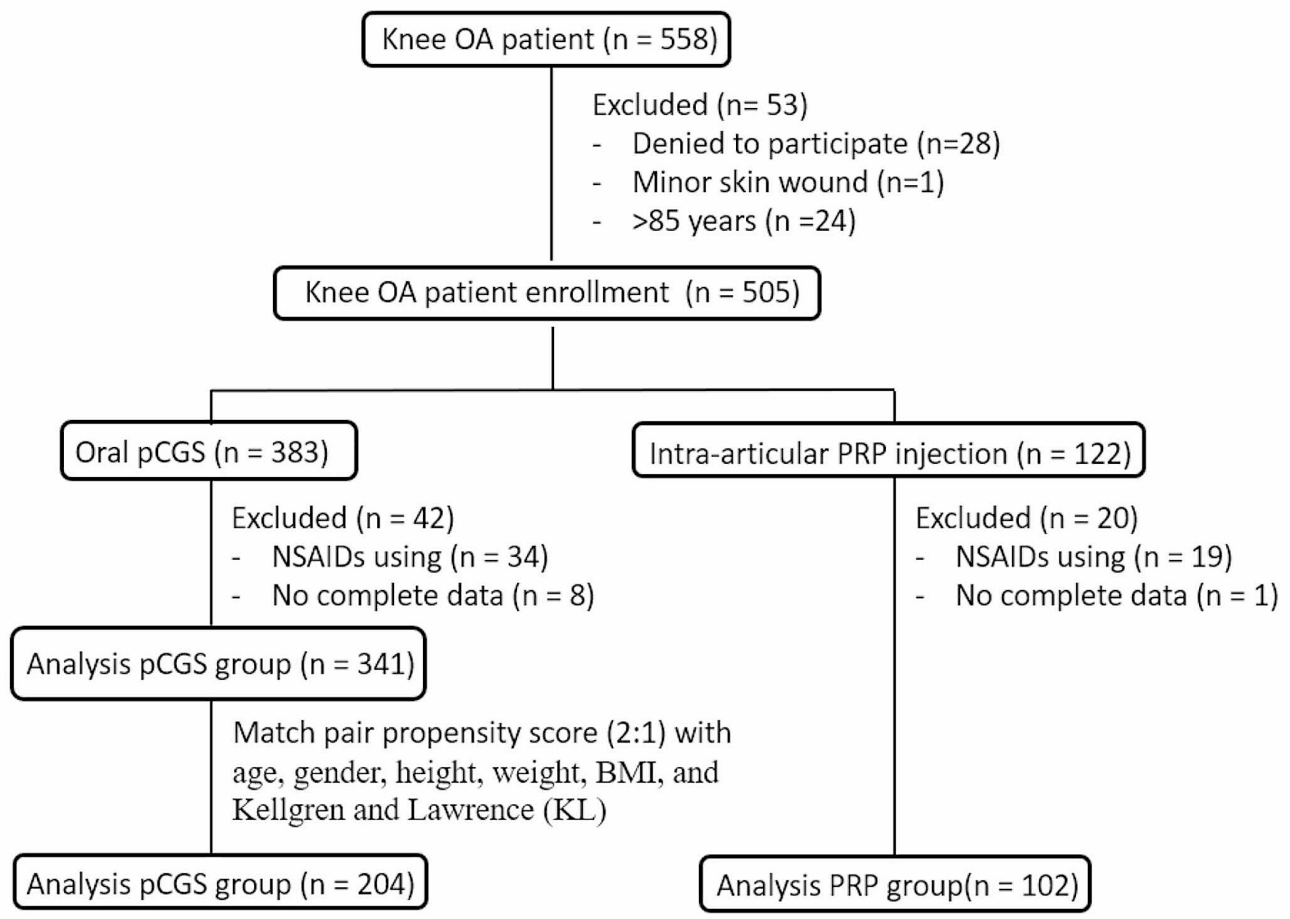
Flow chart of the investigation of knee OA patients with propensity score matching at a ratio of 2:1

Among the 558 eligible patients who met the inclusion criteria, 505 were enrolled in this study. A total of 53 patients were excluded due to reasons such as refusal to participate, minor skin wounds, and age exceeding 85 years. Among the 383 patients treated with oral pCGS, 42 withdrew from the study for similar reasons, and the remaining 341 patients were included in the study. Among the 122 patients who received intra-articular PRP injections, 20 withdrew from the study due to NSAID use or incomplete FU, and the remaining 102 patients were included in the study. Propensity score matching was subsequently performed at a ratio of 2:1, consisting of 204 patients with oral pCGS and 102 patients with PRP. The study flow chart is shown in Fig. 2. The patients’ demographic data are presented in Table 1. There were no significant differences in age, sex, side, height, weight, BMI, or severity of knee OA between the two groups. There were no adverse events during treatment in either group.

**Table 1:** Demographic data of the studied groups

	All patients (*n* = 306)	pCGS group (*n* = 204)	PRP group (*n* = 102)	*p* value
Age (years) (Mean ± SD)	67.5 ± 7.2	67.5 ± 6.7	67.5 ± 8.2	*0.95*
Sex				
- Female (Number, Percent)	281 (91.8%)	187 (91.7%)	94 (92.2%)	*0.88*
- Male (Number, Percent)	25 (8.2%)	17 (8.3%)	8 (7.8%)	
Side				
- Right	171 (55.9%)	113 (55.4%)	58 (56.9%)	*0.81*
- Left	135 (44.1%)	91 (44.6%)	44 (43.1%)	
Height (cm.) (Mean ± SD)	156.7 ± 5.8	156.5 ± 5.6	157.1 ± 6.1	*0.34*
Weight (kg) (Mean ± SD)	63.8 ± 9.6	63.8 ± 9.8	63.9 ± 9.1	*0.48*
BMI (kg/m^2^) (Mean ± SD)	26.0 ± 3.7	26.1 ± 3.8	25.9 ± 3.3	*0.12*
KL classification (Number, Percent)				
- KL 1	59 (19.3%)	35 (17.2%)	24 (23.5%)	*0.18*
- KL 2	101 (33.0%)	71 (34.8%)	30 (29.4%)	
- KL 3	93 (30.4%)	67 (32.8%)	26 (25.5%)	
- KL 4	53 (17.3%)	31 (15.2%)	22 (21.6%)	

pCGS: Patented crystalline glucosamine sulfate

PRP: Platelet-rich plasma

KL: Kellgren and Lawrence

BMI: Body mass index

In both groups, all functional performance outcomes (TUGT, 5xSST, and 3MWDT) improved gradually from baseline to the 1-year FU. Specifically, in the pCGS group, at the 6-week FU, there was a significant improvement in the TUGT outcome, whereas at the 12-week, 24-week, and 1-year FUs, there were significant improvements in all performance tests (TUGT, 5xSST, and 3MWDT), as shown in Table 2. In the PRP group, at the 6-week FU, there were significant improvements in the TUGT and 5xSST outcomes. AT the 12-week, 24-week, and 1-year FUs, there were significant improvements in all of the function performance tests, as shown in Table 2.

**Table 2:** Comparisons between baseline and follow-up functional performance outcomes of the patented crystalline glucosamine sulfate group (pCGS) and platelet-rich plasma (PRP) groups

Functional performance tests	pCGS group (*n* = 204)		PRP group (*n* = 102)	
	Mean ± SD	*p value*	Mean ± SD	*p value*
Five-time sit-to-stand test (sec)				
- Baseline	17.5 ± 6.7		18.7 ± 5.1	
- 6 weeks	16.7 ± 5.9	*0.20*	16.9 ± 4.9	0.01*
- 12 weeks	15.8 ± 5.0	0.003*	16.1 ± 5.0	< 0.001*
- 24 weeks	15.2 ± 4.6	< 0.001*	15.4 ± 3.5	< 0.001*
- 1 year	15.0 ± 4.7	< 0.001*	15.5 ± 3.5	< 0.001*
Time up and go test (sec)				
- Baseline	8.3 ± 2.8		8.9 ± 2.4	
- 6 weeks	7.8 ± 2.3	0.04*	8.2 ± 2.1	0.04*
- 12 weeks	7.5 ± 2.2	< 0.001*	7.7 ± 1.8	< 0.001*
- 24 weeks	7.3 ± 1.9	< 0.001*	7.3 ± 1.3	< 0.001*
- 1 year	7.2 ± 2.1	< 0.001*	7.4 ± 1.6	< 0.001*
3-minute walk distance test (m)				
- Baseline	171.8 ± 34.1		165.9 ± 33.5	
- 6 weeks	174.3 ± 33.3	*0.44*	169.6 ± 28.1	*0.39*
- 12 weeks	178.6 ± 33.2	0.04*	176.4 ± 30.9	0.002*
- 24 weeks	179.1 ± 30.9	0.02*	178.8 ± 25.8	< 0.002*
- 1 year	179.4 ± 29.4	0.02*	177.4 ± 24.0	< 0.001*

pCGS: Patented crystalline glucosamine sulfate

PRP: Platelet-rich plasma

*: Statistically significant

The baseline values of all three functional performance outcomes and FU values at six weeks, 12 weeks, 24 weeks, and one year were not different between groups, as shown in Table 3. However, the change values of all performance tests in the PRP group were greater than those in the pCGS group from the 6-week to the 1-year FUs, with a significant difference found only for the 5xSST at the 6-week FU, as shown in Table 4.

**Table 3:** Functional performance outcomes from baseline to follow-up: comparisons between the patented crystalline glucosamine sulfate group (pCGS) and the platelet-rich plasma group (PRP)

	pCGS group Mean ± SD *(n = 204)*	PRP group Mean ± SD *(n = 102)*	*p* value
Five-time sit-to-stand test (sec)			
- Baseline	17.5 ± 6.7	18.7 ± 5.1	*0.13*
- 6 weeks	16.7 ± 5.9	16.9 ± 4.9	*0.79*
- 12 weeks	15.8 ± 5.0	16.1 ± 5.0	*0.59*
- 24 weeks	15.2 ± 4.6	15.4 ± 3.5	*0.66*
- 1 year	15.0 ± 4.7	15.5 ± 3.5	*0.36*
Time up and go test (sec)			
- Baseline	8.3 ± 2.8	8.9 ± 2.4	*0.09*
- 6 weeks	7.8 ± 2.3	8.2 ± 2.1	*0.14*
- 12 weeks	7.5 ± 2.2	7.7 ± 1.8	*0.36*
- 24 weeks	7.3 ± 1.9	7.3 ± 1.3	*0.88*
- 1 year	7.2 ± 2.1	7.4 ± 1.6	*0.42*
3-minute walk distance test (m)			
- Baseline	171.8 ± 34.1	165.9 ± 33.5	*0.15*
- 6 weeks	174.3 ± 33.3	169.6 ± 28.1	*0.22*
- 12 weeks	178.6 ± 33.2	176.4 ± 30.9	*0.59*
- 24 weeks	179.1 ± 30.9	178.8 ± 25.8	*0.95*
- 1 year	179.4 ± 29.4	177.4 ± 24.0	*0.55*

pCGS: Patented crystalline glucosamine sulfate

PRP: Platelet-rich plasma

**Table 4:** Changes in functional performance outcome from baseline to follow-up: comparisons between patented crystalline glucosamine sulfate (pCGS) and platelet-rich plasma (PRP) groups

	pCGS group (*n* = 204)	PRP group (*n* = 102)				
	∆ from baseline	∆ from baseline		*p value*		
Five-time sit-to-stand test (sec)						
- Baseline						
- 6 weeks	0.8 ± 3.9	1.8 ± 3.6		0.04*		
- 12 weeks	1.7 ± 5.2	2.6 ± 4.1		*0.17*		
- 24 weeks	2.4 ± 5.1	3.3 ± 4.4		*0.12*		
- 1 year	2.5 ± 5.6	3.2 ± 4.8		*0.31*		
Time up and go test (sec)						
- Baseline						
- 6 weeks	0.5 ± 1.8	0.7 ± 1.5		*0.46*		
- 12 weeks	0.8 ± 2.3	1.2 ± 2.3		*0.26*		
- 24 weeks	1.0 ± 2.6	1.6 ± 1.8		*0.07*		
- 1 year	1.2 ± 2.7	1.5 ± 2.4		*0.25*		
3-minute walk distance test (m)						
- Baseline						
- 6 weeks	2.6 ± 24.0	3.7 ± 21.3		*0.68*		
- 12 weeks	6.8 ± 24.7	10.5 ± 25.7		*0.22*		
- 24 weeks	7.3 ± 26.7	12.9 ± 20.2		*0.06*		
- 1 year	7.7 ± 26.4	11.5 ± 23.0		*0.21*		

pCGS: Patented crystalline glucosamine sulfate

PRP: Platelet-rich plasma

*: Statistically significant

## Discussion

To the best of our knowledge, the present study is the first to compare pCGS and PRP for the treatment of knee OA in terms of the functional performance outcomes. Using propensity score matching with a 2:1 ratio based on age, sex, height, weight, and KL classification parameters, there were no significant differences in demographic data between the pCGS or PRP groups. The PRP group showed greater improvements in all functional performance outcomes, including the 5xSST, TUGT, and 3MWDT, from 6-week to 1-year FUs; however, a significant difference was found only for the 5xSST at the 6-week FU.

Although both pCGS and intra-articular PRP are optional nonsurgical treatments for knee OA, most major guidelines from different medical societies involving the treatment of knee OA, including the Osteoarthritis Research Society International (OARSI) [21], the American College of Rheumatology (ACR) [22], and the American Academy of Orthopaedic Surgeons (AAOS) [23, 32], do not or recommend using both treatment options. In contrast, the 2019 ESCEO guidelines for knee OA treatment documented the benefits of pCGS and recommended it as the baseline step of nonsurgical treatment [16]. However, ESCEO does not provide any opinion on PRP or stem cell treatment.

Glucosamine in any chemical form has been acknowledged as a safe option for treating knee OA, as it causes fewer adverse effects than NSAIDs or other painkillers. Among the different forms of glucosamine, studies related to pCGS have shown evidence of benefits over any other form, with very few adverse events [7, 8, 11]. According to reports on pain relief and positive outcomes in functional performance with easy administration and safety after pCGS treatment, this agent has become reimbursable for treating knee OA in some countries, such as Thailand and other Southeast Asian countries [33, 34], where the increasing usage of pCGS has raised concerns about its efficacy and cost-effectiveness. Recent studies have shown that pCGS is cost effective and is worth considering for use in the first step of treatment for knee OA [35, 36].

Amarase et al. reported improved pain symptoms after pCGS treatment related directly to increased functional performance, including TUGT from 6 weeks and 5xSST from 12 weeks [33]. Therefore, in addition to pain symptoms, functional performance tests help evaluate the actual clinical improvement of patients during treatment without patient bias related to specific preferences. In the present study, outcomes on the three performance tests (5xSST, TUGT, and 3 MWDT) gradually improved, with significant improvements observed after 12 weeks. This could be explained by the fact that pCGS is a primary substrate for synthesizing a cartilage matrix via oral administration; clinical improvement, especially performance, could take several weeks. Additionally, continuous treatment with pCGS in the present study showed that it positively affected functional performance over one year.

PRP treatment has recently demonstrated positive results and has become well accepted among knee OA patients worldwide, even though most major guidelines do not include or recommend it [21, 22, 32]. During the past ten years, this optional treatment, although it requires an invasive procedure (blood drawn and intra-articular injection), has become a popular, desirable, nonsurgical treatment for knee OA patients [26, 37, 38]. Studies on PRP applied in knee OA, with various protocols of preparation and injection, have shown that it improves patient clinical outcomes by releasing several growth factors and cytokines, promoting repair and minimizing the inflammation that occurs in the process of degenerative arthritis [39–43]. Some studies reported better pain relief and improved functional outcomes with PRP than with placebo control, hyaluronic acid, or steroid injection [44, 45]. Although leukocyte-rich PRP (LR-PRP) is an optional PRP preparation for treating knee OA, a recent meta-analysis and systematic review supported the use of LP-PRP in terms of a lower incidence of adverse events than LR-PRP [46].

The results of the PRP group in the present study reported similar improvements in the TUGT, 5xSST, and 3MWDT outcomes to those reported by Ngarmukos et al. [47], with extension until the 1-year FU. PRP carries several orthobiologic agents with intra-articular delivery. Therefore, the mechanism of action to provide functional improvement should be faster than that of the oral pCGS, and the changes in the outcomes of the performance tests should be greater in the PRP group than the oral pCGS group, especially in the early phase of treatment.

Despite the findings that the improvements in functional performance in the PRP group were significantly greater than those observed in the pCGS group, both treatment groups provided similar performance-based outcomes until the one-year FU. With respect to the invasiveness of treatment, PRP requires a particular body-invasive procedure for preparation. Therefore, the oral administration of pCGS may be an alternative noninvasive treatment.

There are several limitations in this study. First, the study design was not a randomized controlled trial, which might affect the proper sample allocation of both groups. However, propensity score matching was employed with a 2:1 ratio (pCGS: PRP) based on age, sex, height, weight, and KL classification parameters to control for the similarity of the samples in both groups, and the demographic data were not significantly different between the groups. Second, the outcomes of this study focused solely on functional performance outcomes and did not include other aspects, such as pain, the WOMAC, or the SF-12. However, this study was designed to examine functional performance evaluation, which provided more evidence-based data in optional treatment in which the patient might have a positive bias, especially both pCGS and PRP. Focusing on functional performance outcomes could provide insight into the quality of a patient’s daily activities. Finally, blinding the treatment method to the patients in the two groups was not possible because of the different methods of treatment administration, which might introduce patient bias. However, the collection of functional performance outcomes is an objective measure that may be less prone to bias than other subjective outcomes are.

## Conclusions

Although PRP provided significantly faster improvement in 5xSST outcomes than did pCGS, both optional treatments were effective for treating knee OA, as evidenced by improved functional performance outcomes until one year without harm. The procedure for using PRP for treatment is more invasive and complicated than the use of oral pCGS, and it is important to weight the benefits and drawbacks of PRP over pCGS to treat knee OA.

## Data Availability

No datasets were generated or analysed during the current study.
